# Epilepsy-Associated UBE3A Deficiency Downregulates Retinoic Acid Signalling Pathway

**DOI:** 10.3389/fgene.2021.681295

**Published:** 2021-04-28

**Authors:** Meimiao Fang, Yali Li, Jin Ren, Ronggui Hu, Xiaobo Gao, Liang Chen

**Affiliations:** ^1^School of Medicine, Guizhou University, Guiyang, China; ^2^State Key Laboratory of Molecular Biology, Shanghai Institute of Biochemistry and Cell Biology, Center for Excellence in Molecular Cell Science, Chinese Academy of Sciences, Shanghai, China; ^3^University of Chinese Academy of Sciences, Beijing, China; ^4^Institute for Translational Brain Research, State Key Laboratory of Medical Neurobiology and MOE Frontiers Center for Brain Science, Fudan University, Shanghai, China; ^5^Department of Neurosurgery, Huashan Hospital, Fudan University, Shanghai, China; ^6^Shanghai Key Laboratory of Brain Function Restoration and Neural Regeneration, Shanghai Clinical Medical Center of Neurosurgery, MOE Frontiers Center for Brain Science, Shanghai Medical College, Fudan University, Shanghai, China

**Keywords:** UBE3A, RA signalling pathway, RARα, coactivator, Angelman Syndrome

## Abstract

Ubiquitin-protein ligase E3A (UBE3A) has dual functions as a E3 ubiquitin-protein ligase and coactivator of nuclear hormone receptors. Mutations or deletions of the maternally inherited UBE3A gene cause Angelman syndrome. Here, we performed transcriptome profiling in the hippocampus of *Ube3a^*m*+/p+^* and *Ube3a^*m–/p*+^* mice, and determined that the expression of the retinoic acid (RA) signalling pathway was downregulated in Ube3a-deficient mice compared to WT mice. Furthermore, we demonstrated that UBE3A directly interacts with RARα and may function as a coactivator of the nuclear receptor RARα to participate in the regulation of gene expression. Loss of UBE3A expression caused the downregulation of the expression of RA-related genes, including *Erbb4, Dpysl3, Calb1, Pten*, and *Arhgap5* in Ube3a^*m–/p*+^ mice brain tissues. This work revealed a new role for UBE3A in regulating retinoic acid (RA) signalling downstream genes and hopefully to shed light on the potential drug target of AS.

## Introduction

The UBE3A (also known as E6AP) gene is located on the proximal arm of the 15th chromosome at the q11–q13 site in humans (Kishino and Wagstaff, 1998). The UBE3A gene encodes three isoforms: UBE3A-isoform I, UBE3A-isoform II, and UBE3A-isoform III. UBE3A exerts two independent functions *in vivo*, with the HECT domain mainly functioning as a ubiquitin ligase, with LxxLL motif as a nuclear receptor coactivator (Khan et al., 2006; Wang et al., 2017). The LxxLL motif is a highly conserved signature sequence that binds nuclear receptors to activate gene expression. UBE3A is biallelically expressed in most tissues except nerve tissues, where it is imprinted with maternal allelic expression (Yamasaki et al., 2003). In most neurons, UBE3A is expressed only from the maternal allele, while the paternal allele is epigenetically silent (paternal imprinting) (Albrecht et al., 1997). Researchers have identified loss-of-function mutations of maternal UBE3A in 8% Angelman syndrome (AS) cases (Maranga et al., 2020). Angelman syndrome prevalence ranges from approximately 1 in 12,000 to 1 in 20,000 (Buiting et al., 2015). The behavioural characteristics of AS include intellectual disability, seizures, short attention span, excessive exercise behaviour, sleep disturbance, happy disposition and fascination with water (Williams et al., 2006; Tan et al., 2011; Gu et al., 2019).

All-*trans* retinoic acid (RA) is the active metabolite of vitamin A, which regulates the expression of many different genes in embryonic and adult organisms (Ross et al., 2000). RA can activate or repress transcription of key developmental genes, regulates the expression of many different genes in embryonic and adult organisms. At present, more than 600 genes have been reported to respond to RA (Balmer and Blomhoff, 2002; Savory et al., 2014). RA activates gene transcription by interacting with a transcription factor complex that includes RA receptor (RAR) and retinoic acid X receptor (RXR) heterodimers (Mark et al., 2006). The RAR superfamily members include RAR alpha, RAR beta, and RAR gamma; and RXRs superfamily includes RXR alpha, RXR beta, and RXR gamma in mammals. The heterodimer binds to the RARE (RA responding element) sequence and recruits a series of corepressors (NCoR and SMRT) to repress gene transcription in the absence of RA (Bastien and Rochette-Egly, 2004; Balmer and Blomhoff, 2005; Amann et al., 2011). To activate gene expression, RAR-RXR must change the structure of suppressed chromatin in the presence of RA and then dissociate from the corepressor and recruit coactivator to promote the binding of the transcription complex in the promoter region (Egea et al., 2001). RAR alpha (RARα) is commonly found in embryonic and adult tissues, and the *RARα* gene is located on chromosome 17q21. RARα is involved in regulating cell differentiation, embryonic development, vision formation, metabolism and many other life processes. RARα plays a key role in the adult brain, participating in the homeostatic control of synaptic plasticity, which is essential for memory function (Zhu et al., 2010; Bosch et al., 2012; Goncalves et al., 2013).

In our study, we performed RNA-seq on the hippocampus of UBE3A maternal deficient mice and found that loss of UBE3A expression affected many genes involved in the RA signalling pathway, which disruption led to dysregulate of development of many organs and nervous system signalling pathways. We further demonstrated that UBE3A regulates RARE-luciferase expression as a coactivator of the nuclear receptor RARα and directly interacts with RARα. Moreover, loss of UBE3A expression led to dysregulation of *Erbb4, Dpysl3, Calb1, Pten, Arhgap5*. Our results reveal the novel role of UBE3A in the regulation of RA signalling and provide insight into potential therapeutic targets for Angelman syndrome.

## Materials and Methods

### GeneMANIA

GeneMANIA^1^ is a website that provides information about protein and genetic interactions, coexpression, pathways, colocalization, and protein domain similarity of the submitted genes (Warde-Farley et al., 2010). GeneMANIA generates a list of genes with similar functions to the query gene and constructs an interactive functional-association network to illustrate relationships between genes and datasets.

### Cell Culture and Transfection

HEK-293T (Life Technologies), SH-SY5Y (ATCC), H1299 (ATCC), and mouse embryonic fibroblast (MEF) cells were maintained in DMEM (Corning) supplemented with 10% FBS (Gibco) and 50 μg/mL penicillin/streptomycin (Life Technologies). MEF cells were isolated at embryonic day 11.5. All cells were cultured in a humidified 5% CO_2_ air incubator at 37°C. Plasmid transfections into SH-SY5Y cells and HEK-293T cells were achieved with polyethylenimine (PEI) (Sigma-Aldrich, United States). H1299 cells were transfected with Lipofectamine 2000 (Life Technologies, United States) according to the manual.

### Plasmid Construction

Briefly, restriction enzyme digestion and ligation reactions (NEB) were performed using traditional cloning methods. In this work, human *UBE3A* (iso1 unless otherwise indicated) and *RARα* were used as the initial templates, and the indicated plasmids were constructed on different backbones. Point mutations of the indicated plasmids were generated by site-directed mutagenesis. The following plasmids were constructed in this work: pGEX4T-1-UBE3A-GST, pCDNA3.0-Flag-UBE3A, pCDNA3.0-Myc-UBE3A, pCDNA3.0-Myc-UBE3A(iso2), pCDNA3.0-Myc-UBE3A(iso3), pCDNA3.0-Myc-UBE3A-C843A, pCDNA3.0-RARα-HA, pRK5-His6-UB, pCDNA3.0-Myc-UBE3A-L260A/V261A(L1A), pCDNA3.0-Myc-UBE3A-L412A/L413A(L2A), pCDNA3.0-Myc -UBE3A-V457A/L458A(L3A), pCDNA3.0-Myc-UBE3A-L513A/V514A(L4A), pCDNA3.0-Myc-UBE3A-L665A/L666A(L5A), and pCDNA3.0-Myc-UBE3A-L260A/V261A/L412A/L413A/V457A/L458A/L513A/V514A/L665A/L666A(LA).

### Reagents

All-*trans-*retinoic acid was obtained from Sigma-Aldrich (R2625).

### RNA Sequencing

RNA samples were extracted from the hippocampal tissues of 6- to 8-week-old mice using an RNA simple total RNA kit (Tiangen, China) and were subjected to high-throughput sequencing. The minimum clean data size of each group was 6 GB.

### Luciferase Assay

To generate the pGL4.22-RARE luciferase construct, 3 × RARE (retinoic acid responsive elements) was cloned into the pGL4.22 vector (Promega). RARE-specific luciferase reporter assays were performed using VP-SFM (Gibco). After treatment with 1 μM RA for 8 h, the total luciferase activities of the cultured cells were measured following the manufacturer’s instructions (Promega).

### Quantitative Real-Time PCR (qPCR)

Total RNA was extracted using the RNA simple Total RNA Extraction Kit (DP-419, TIANGEN), and then total RNA was reverse transcribed into cDNA using ReverTra Ace qPCR RT Master Mix with gDNA Remover (FSQ-301, TOYOBO). Cham Q Universal SYBR qPCR Master Mix (Vazyme, China) was used to perform RT-qPCR. All primers in this work are shown in Supplementary Table 6. The relative expression values of selected genes were calculated using the 2^–Δ^ ^Δ^ ^*Ct*^ method and normalized to the *Gapdh* relative expression values.

### Immunofluorescence

Brains were obtained from 8-week-old mice, fixed with 4% paraformaldehyde, and immersed in PBS supplemented with 30% sucrose (Sigma-Aldrich, United States) until setting. Coronal sections of the brain were obtained using a Lecia CM3050 S Research Cryostat (Leica Biosystems). The brain slices were treated with 0.3% Triton X-100 for 15 min at room temperature and then blocked with 3% normal goat serum (Boster, China). Ube3a and Erbb4 were visualized by staining with rabbit monoclonal Ube3a (7526S, CST, 1:200 dilution) and mouse monoclonal Erbb4 (sc-8050, Santa Cruz, 1:50 dilution) primary antibodies, followed by FITC-conjugated rabbit secondary antibody and Cy3-conjugated mouse secondary antibody, respectively. The brain sections were counterstained with DAPI for nuclear staining. Fluorescent images were obtained using an Olympus FV1200 confocal microscope (Olympus).

### Co-immunoprecipitation Assay

HEK-293T cells with endogenous or exogenous protein expression were lysed in Co-IP buffer (50 mM Tris–HCl, pH 7.5, 150 mM NaCl, 5 mM EDTA, 1% NP-40) with proteasome inhibitor cocktail (Roche, Switzerland). Cell pellets were sonicated and centrifuged at 15,000 rpm for 10 min at 4°C. The supernatant was incubated with specific antibody beads or protein G agarose beads overnight at 4°C. The beads were washed with Co-IP buffer five times. Then, the recovered beads were boiled in 2 × SDS-PAGE loading buffer, and the indicated antibodies were used for immunoblotting analysis. The primary antibodies were as follows: anti-Flag (F1804, Sigma-Aldrich, 1:2,000 dilution), anti-HA (H6908, Sigma-Aldrich, 1:2,000 dilution), anti-actin (A2228, Sigma-Aldrich, 1:8,000 dilution), anti-UBE3A (sc-166689, Santa Cruz, 1:500 dilution), anti-RARα (ab275745, Abcam, 1:1,000 dilution), anti-GAPDH (sc-32233, Santa Cruz, 1:4,000 dilution), anti-His (66005-1lg, Proteintech, 1:1,000 dilution), anti-Myc (67447-1-lg, Proteintech, 1:1,000 dilution), anti-GST(66001-1-lg, Proteintech, 1:5,000 dilution).

### Ubiquitination Assay

HEK-293T cells transiently transfected with the indicated plasmids were lysed with IP buffer (50 mM Tris–HCl, pH 7.5, 150 mM NaCl, 5 mM EDTA, 1% NP-40, 0.1% SDS, 0.5% sodium deoxycholate) with a proteasome inhibitor cocktail. The precipitation process was performed as described above. The beads were washed three times with IP buffer and subjected to immunoblotting.

### Expression and Purification of Recombinant Proteins

GST-RARα- and UBE3A-His-tagged proteins were expressed in *E. coli* (BL21) cells. The expression of tagged proteins was induced by incubation with 400 mM IPTG for 16 h at 16°C. The cells were pelleted, lysed in PBS (phosphate-buffered saline) buffer and incubated with glutathione beads (GE, United Kingdom) or Ni-NTA agarose beads (Qiagen, United States) to enrich the respective proteins. The bound proteins were eluted with 20 mM reduced L-glutathione or 300 mM imidazole dissolved in PBS buffer (pH 8.0) and then dialyzed in PBS buffer supplemented with 20% glycerol. The purified proteins were aliquoted and stored at −80°C.

### GST Pull-Down Assay

Purified recombinant GST-RARα and UBE3A-His proteins and Glutathione Sepharose 4B beads (GE) were incubated in 500 μl pull-down buffer (50 mM Tris–HCl, pH 8.0, 200 mM NaCl, 1 mM EDTA, 1% NP-40, 1 mM DTT, 10 mM MgCl2) for 2 h at 4°C. The beads were washed three times with pull-down buffer, resuspended in 2 × SDS-PAGE loading buffer and then subjected to western blotting.

### Western Blotting

Total protein was dissolved in SDS loading buffer, boiled for 10 min, and subjected to SDS-PAGE. The proteins were transferred to polyvinylidene difluoride membranes (Millipore, Bedford, MA, United States). The membranes were blocked in 10% fat-free milk for 1 h at RT and incubated with primary antibodies in primary antibody dilution buffer (Beyotime, China) overnight at 4°C. Then, the membranes were further incubated with the corresponding secondary antibody at RT for 1 h and washed three times with TBST. The protein bands were visualized using Quantity One Software (Bio-Rad, United States) after incubation with enhanced chemiluminescence reagent (Millipore).

### Animals

Angelman syndrome (Ube3a^*m*–/p+^) mice were kindly provided by Dr. Zhiqi Xiong, Chinese Academy of Sciences, Shanghai. All mice involved in the study were in the C57BL/6 background (SLAC, China). All animal experiments strictly followed the instructions of the Institutional Animal Care and Use Committee (IACUC) at the Center for Excellence in Molecular Cell Science, CAS.

### Statistical Analysis

Three independent experiments were performed, and the results are expressed as the mean ± standard error of the mean (SEM). Data were compared using unpaired Student’s *t* tests and ordinary one-way ANOVA in GraphPad Prism 8 software (GraphPad Software). A *P* value < 0.05 was considered statistically significant and is indicated as follows: ^∗^*P* < 0.05, ^∗∗^*P < 0.01*, ^∗∗∗^*P < 0.001*, ^****^P < 0.0001.

### Accession Number

The RNA-seq data have been deposited in the Gene Expression Omnibus database at NCBI under accession number GSE168889.

## Results

### Loss of UBE3A Expression Affects the RA Signalling Pathway

**FIGURE 1:**
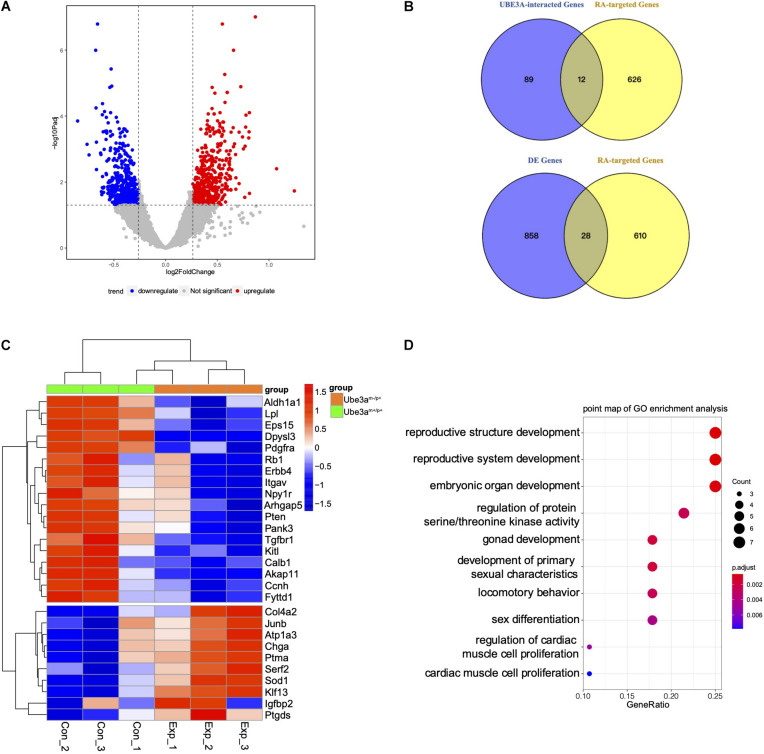
Gene expression profiling in the hippocampus of mice. **(A)** Volcano plot analysis from the hippocampus of Ube3a^*m–/p*+^ mice and Ube3a^*m*+/p+^ mice (Foldchange > 1.2; *P* < 0.05). **(B)** Venn analysis was used for enrichment between the most related to UBE3A and RA-regulated genes or differentially expressed genes. **(C)** Comparison of convergent differential RA-targeted genes expression between Ube3a^*m*+/p+^ and Ube3a^*m–/p*+^ mice. The mean numbers of reads were used. Mouse samples were divided into two groups: Ube3a^*m*+/p+^ mice (*n* = 3) and Ube3a^*m–/p*+^ mice (*n* = 3). **(D)** Enriched Biological Process pathway in GO analysis (*P* < 0.05).

As a versatile protein, UBE3A participates in many processes involving in the nervous development and plasticity. However, little mechanistic insight into the role of UBE3A in RA signalling has been revealed. In this work, to determine whether UBE3A is associated with RA signalling pathway, first, the UBE3A regulatory network was constructed using the GeneMANIA web-based utility. A total of 101 genes were identified most related to UBE3A involved in physical interactions, co-expression, colocalization, pathway, genetic interactions, shared protein domains and others (Supplementary Figure 1 and Supplementary Table 1). Meanwhile, we collected 628 RA-targeted genes from the literature (Supplementary Table 3). There were total 12 overlapping genes between UBE3A network and RA-targeted genes (Figure 1B and Supplementary Table 4), indicating UBE3A may play a role in regulation RA signalling pathway. The hippocampus, which is located in the centre of the brain and is of crucial importance in memory regulation, fear condition, anxiety and cognition, is receiving increasing attention in the study of AS (Maranga et al., 2020). Using hippocampal tissues obtaining from Ube3a^*m–/p*+^ mice and Ube3a^*m*+/p+^ mice, we performed whole-genome transcriptome profiling to characterize the differentially expressed genes. A total of 886 genes were screened with the threshold of significance at *P* < 0.05 and foldchange > 1.2, among which 463 genes were up-regulated and 423 genes were down-regulated in the Ube3a^*m–/p*+^ group compared to WT control group, shown in volcano plot (Figure 1A and Supplementary Table 2). To explore the potential role of RA signalling pathway in Ube3a^*m–/p*+^ mice, we specifically selected the convergent genes both in differentially expressed genes and in the RA-targeted genes. We found that 28 RA-targeted genes are differentially expressed in Ube3a^*m–/p*+^ male mice compared to WT male mice (Figure 1B). The detailed results were displayed in heatmap. Compared to Ube3a^*m–/p*+^ mice from WT mice, most genes exhibited lower expression including *Erbb4, Dpysl3, Calb1, Pten, Arhgap5* (Figure 1C). In order to better understand the potential functions of convergent differentially expressed genes, Gene Ontology (GO) enrichment analysis was carried out to assess the involved pathways. Biological process pathway in GO analysis results showed that multiple development pathway was enriched and the locomotory behaviour which abnormal is common AS patients was also enriched (Figure 1D). The results showed that UBE3A is closely related to the RA signalling pathway, and dysregulated RA signalling may play a role in the pathogenesis in AS.

### UBE3A Regulates RARE-Luciferase Reporter Expression as a Coactivator of RARα

**FIGURE 2:**
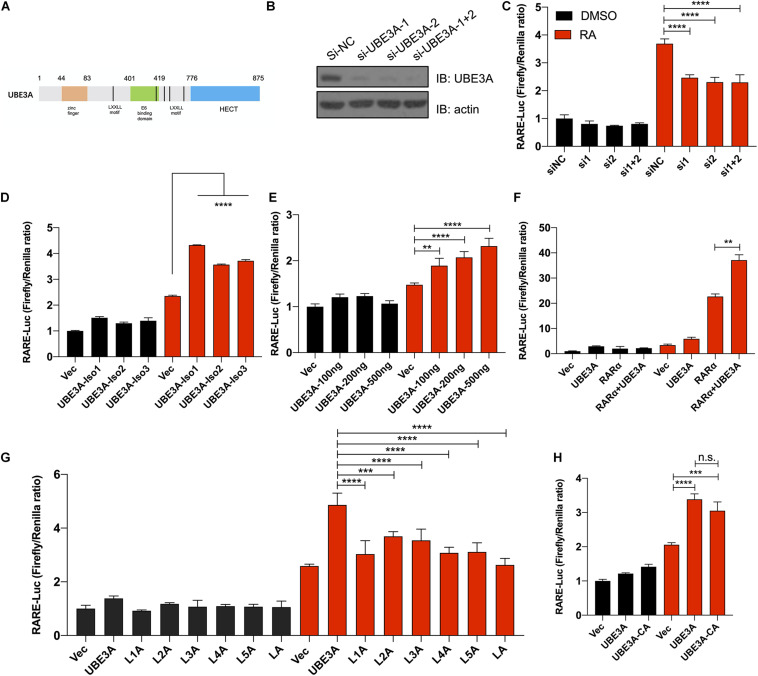
UBE3A acts as a coactivator regulating RARE-luciferase expression. **(A)** Schematic structure of UBE3A protein. UBE3A is an 862-amino acid protein that mainly includes five LxxLL domains, a zinc finger domain and a HECT domain. **(B)** Endogenous UBE3A protein was knocked down upon siRNA treatment. siRNA-UBE3A1, siRNA-UBE3A2 and negative control were transfected into H1299 lung cancer cells, and the protein expression of UBE3A was assessed by western blotting. **(C)** UBE3A knockdown decreased luciferase activities. H1299 cells were transfected with siUBE3As (the indicated siRNA sequences are listed in Supplementary Table 5), and RARE luciferase activities were tested after stimulation with 1 μM ATRA for 8 h. **(D)** All isoforms of UBE3A showed activation of RARE-luciferase activities. H1299-KO-UBE3A cells were transfected with vector or different isoforms of UBE3A and then treated with 1 μM ATRA for 8 h. **(E)** UBE3A could activate luciferase activities in a dose-dependent manner. SH-SY5Y cells were transfected with the indicated dosages of UBE3A followed by 1 μM ATRA treatment for 8 h, and the luciferase ratios were detected. **(F)** UBE3A and RARα showed coactivation of RARE-luciferase activities. H1299-KO-UBE3A cells were transfected with vector, UBE3A, RARα or UBE3A + RARα and then treated with 1 μM ATRA for 8 h. **(G)** Mutant in the LxxLL domain compromised the activation of UBE3A. H1299-KO-UBE3A cells were transfected with the indicated LxxLL mutation (L1A or L2A or L3A or L4A or L5A or LA, labelled in method 2.2) followed by 1 μM ATRA treatment for 8 h. **(H)** The ligase mutant C843A mutant did not affect luciferase activity. Wild-type UBE3A and ligase mutants were overexpressed in H1299-KO-UBE3A cell lines followed by 1 μM ATRA treatment for 8 h. The normalized luciferase activities represent ratios of firefly to Renilla values. The values are expressed as the means ± SEM from at least three independent repetitions. *P < 0.05, **P < 0.01, ***P < 0.001, ****P < 0.0001. **(C–H)** one-way ANOVA with Bonferroni *post hoc* test.

Previous gene enrichment analysis and RNA-seq data indicated that the absence of UBE3A may affect the RA signalling pathway. We constructed a RARE-luciferase reporter system to directly detect the transcriptional activity of the RA signalling pathway. In H1299 cells, upon the knockdown of endogenous UBE3A expression, RARE-luciferase activities were significantly inhibited with RA treatment (Figures 2B,C). Using the CRISPR-Cas9 method, we constructed a UBE3A-knockout H1299 cell line to avoid interference from endogenous proteins. There are three isoforms of UBE3A *in vivo*. When all three isoforms were overexpressed in H1299 UBE3A KO cells, the RARE-luciferase results indicated that all three isoforms showed activation with RA stimulation compared to the vector (Figure 2D). Considering that UBE3A acts as a coactivator, we wondered whether the activation effects could increase with increasing protein levels. To this end, increasing doses of UBE3A were overexpressed in H1299 KO cells, and the luciferase results indicated that the activation positively correlated with the protein dosages (Figure 2E).

Previous studies have demonstrated that UBE3A can act as a coactivator in the hormone signalling pathway (Ramamoorthy and Nawaz, 2008). We hypothesized that UBE3A may act as a coactivator to activate RA downstream genes. The UBE3A protein has five predicted coactivator domains located mainly in the middle region of the protein structure, with the amino acid sequence LxxL/VL (L represents leucine residue, x represents any residue, V represents valine residue) (Figure 2A). RARα is an important receptor of retinoic acid that binds all-*trans* and 9-*cis* RA. Our experiments show that UBE3A regulates RARE-luciferase expression by acting as a coactivator of RARα (Figure 2F). Overall, these results suggested that UBE3A could regulate RA signalling as a coactivator.

Then, to determine the involvement of the putative LxxLL motif in the regulation process, we constructed six different mutants referred to as UBE3A^*L260A/V261A*^, UBE3A^*L412A/L413A*^, UBE3A^*V457A/L458A*^, UBE3A^*L513A/V514A*^, UBE3A^*L665A/L666A*^, and UBE3A^*L260A/V261A/L412A/L413A/V457A/L458A/L513A/V514A/L665A/*^ ^*L*666*A*^. When individual mutant plasmids were overexpressed in H1299-KO-UBE3A cells, the RARE-luciferase results showed that a single UBE3A LxxLL motif mutant could decrease UBE3A coactivator activity compared to the UBE3A wild-type group, with all mutants almost losing activation activity (Figure 2G). UBE3A could function as a ubiquitin ligase; thus, we wondered whether ubiquitin ligase activity was involved in regulating the RA signalling pathway. Therefore, an E3 ubiquitin ligase-dead mutant UBE3A^*C384A*^ was constructed, and the results showed that the luciferase activity was not significantly changed (Figure 2H). Together, these data indicated that UBE3A regulates RA signalling in a LxxLL-dependent and E3 ligase-independent manner.

### Human UBE3A Interacts With Nuclear Receptor RARα

**FIGURE 3:**
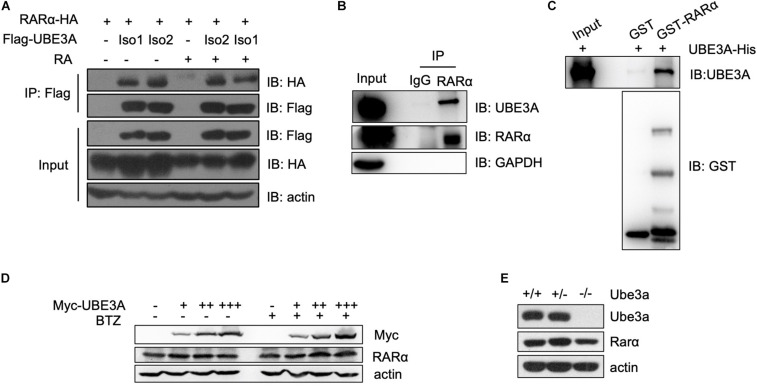
UBE3A directly interacts with RARα. **(A,B)** The interaction of UBE3A and RARα in both endogenous and ectopically expressed HEK-293T cells. Co-immunoprecipitation (Co-IP) was performed for ectopically expressed **(A)** or endogenous **(B)** UBE3A and RARα in HEK-293T cells treated with 1 μM RA or DMSO for 8 h. **(C)** The directly interaction of UBE3A and RARα. GST pull-down assays were performed with GST or GST-RARα and UBE3A-His. **(D,E)** Endogenous RARα **(D)** or Rarα **(E)** remained unchanged upon different dosages of UBE3A **(D)** in HEK-293T cells or Ube3a **(E)** protein levels in MEFs.

In the RA signalling pathway, RARα is a receptor that responds to retinoid acid and exerts a transcriptional regulatory function (Maranga et al., 2020). We tested whether UBE3A affects the RA signalling pathway through interaction with the nuclear receptor RARα. Coimmunoprecipitation assays showed that both endogenous and ectopically expressed UBE3A and RARα proteins could form a complex in HEK-293T cells with or without RA treatment (Figures 3A,B). To further assess whether UBE3A is directly associated with RARα, we carried out glutathione S-transferase (GST) pull-down assays. When recombinant GST-fused RARα protein was mixed with His-tagged UBE3A protein, UBE3A protein was efficiently pulled down by RARα, which was immobilized on glutathione-agarose beads (Figure 3C). To determine which truncated fragment of RARα interacts with UBE3A, we purified the truncated protein of RARα and found that it mainly binds to RARα-88-153, which is the DNA binding domain of RARα. These data indicate that UBE3A can bind to RARα mainly through the RARα-88-153 domain (Supplementary Figure 2). To further verify whether UBE3A could conjugate ubiquitination modification on RARα, wild-type UBE3A or its E3 ligase-dead mutant (C843A) mutant was ectopically expressed in HEK-293T cells. Immunoprecipitation results showed that UBE3A did not further increase the ubiquitylation level of RARα compared to the vector or ligase-mutant group (Supplementary Figure 2), which correlated with the RARE-luciferase results (Figure 2H). Furthermore, the endogenous RARα protein level remained unchanged upon treatment with different dosages of UBE3A or the proteasome inhibitor BTZ in HEK-293T cells (Figure 3D). Moreover, in MEFs with different Ube3a genotypes, the endogenous Rarα protein level was also almost equal among the groups (Figure 3E). Overall results demonstrated that UBE3A mainly function as a coactivator of RARα rather than E3 ligase to participate regulating in RA signalling pathway.

### Loss of UBE3A Expression Led to Dysregulation of RA Downstream Genes

**FIGURE 4:**
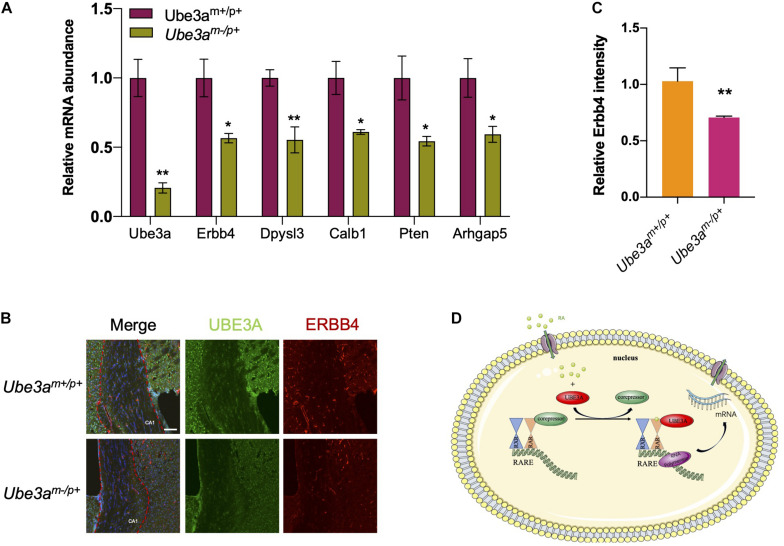
Loss of UBE3A expression leads to the dysregulation of RA-targeted genes. **(A)** Endogenous RA-related genes were reduced in the Ube3a deficient hippocampus. The mRNA levels of target genes were quantitated by real-time qPCR in mouse hippocampal tissues. **(B)** Representative images of Erbb4 staining in hippocampal coronal slices. CA1 region was marked by the red dotted line. Scale bar, 50 μm. **(C)** Quantitated intensities of ERBB4 in the hippocampal region of wild-type and Ube3a deficient mice. **(D)** A working model depicting how UBE3A acts as a coactivator of RARα to participate in the regulation of RA signalling. The values are expressed as the means ± SEM from at least three independent repetitions. *P < 0.05, **P < 0.01, ***P < 0.001. **(B,D)** Unpaired two-tailed *t* test, **(A)** one-way ANOVA with Bonferroni *post hoc* test.

Since UBE3A could function as a coactivator to upregulate RA-related signalling pathways, we wondered whether the neuron-related gene expression pattern would change upon the loss of UBE3A expression in Ube3a^*m*–/p+^ mice. Therefore, we intended to measure the expression levels of genes related to the RA signalling pathway which were also involved in developmental progression. In the hippocampus tissue from Ube3a^*m*–/p+^ mice and wild-type mice, real-time qPCR results showed that *Erbb4, Dpysl3, Calb1, Pten, Arhgap5* levels were decreased upon the loss of *Ube3a* expression (Figure 4A). Immunofluorescence analysis of brain slices showed that ERBB4 expression levels were significantly decreased in CA1 region of hippocampus of Ube3a^*m*–/p+^ mice compared to WT mice (Figures 4B,C). Overall, we found that loss of UBE3A expression in the Ube3a^*m*–/p+^ mouse brain could lead to dysregulation of the endogenous RA downstream gene expression pattern. These data demonstrated that UBE3A could interact with the RA receptor RARα in a non-ubiquitinating manner to participate in RA signalling pathway regulation (Figure 4D).

## Discussion

Recent studies have showed that UBE3A was involved in the etiology of many human tumors and neurological diseases (Sell and Margolis, 2015; Vatsa and Jana, 2018; Maranga et al., 2020). In the present of E6 protein, UBE3A (also known as E6AP) could function as E3 ligase and degrade P53 protein leading to occurrence of cervical cancer (Martinez-Zapien et al., 2016). With hyperfunction or overdosage of UBE3A protein, many synaptic proteins were disrupted, thus causing autism in humans (Greer et al., 2010; Lopez et al., 2018; Sun et al., 2019). However, the coactivator function of UBE3A in the Angelman syndrome was little explored. In current study, we found UBE3A could function as a coactivator in RARE-luciferase reporter assay (Figure 3), confirmed by the network overlap of UBE3A and RA signalling (Figure 1C).

In the broader literature, there is increasing researches showing that disrupted RA signalling pathway might be associated with neurological diseases. Retinoic acid (RA) is a metabolite of retinol (vitamin A), functions as a ligand for nuclear RA receptors (RARs) that regulate development of chordate animals (Ghyselinck and Duester, 2019). RARα is expressed in many tissues and organs and is involved in embryonic development and many other life processes. In current study, we demonstrated that UBE3A could directly interacts with RARα, and facilitate the gene expression regulated by RARα. The protein stability of RARα is rather stable as it stayed almost unchanged upon BTZ treatment (Figure 3D). In one way, it showed that RARα is ready to response to any stimuli efficiently. In another way, it might suggest that RARα might form a complex and undergo other pathway to achieve the protein turnover. Upon loss of UBE3A, the neural development related gene expression levels were compromised (Figure 4A). ErbB4 is a member of the EGF receptor (EGFR) family of RTKs, which are activated by neuregulins and other growth factors on the cell membrane. Like other RTKs, ErbB4 contains an extracellular ligand binding domain which connected to the cytoplasmic portion by a single transmembrane (TM) domain. Nuclear ErbB4 can represses transcription of neuronal differentiation genes. Moreover, NRG1-ErbB4 signalling have a role in neuronal migration, axon guidance, glial cell development, axon myelination and axon ensheathment, Synapse formation. It is possible that the decreased expression of ErbB4 disrupts the developmental processes mentioned above to affects the progression of the Angelman syndrome. Perhaps this can provide us with new insights in the treatment of Angelman Syndrome. Researchers may apply the drugs that increase the overall transcription level to up-regulate the expression of target genes in the RA signalling pathway, which may have a therapeutic benefit.

Angelman syndrome is a neurological disease with extremely complicated mechanisms which many pathways were altered including development of nervous system, neurophysiological activity, neuron morphology and other aspects (Sun et al., 2019; Maranga et al., 2020). Angelman syndrome is a neurodevelopmental disease caused by the loss of function of maternal inherited *UBE3A*, while paternal *UBE3A* remains intact (Maranga et al., 2020). Therefore, current mainstream strategy for AS treatment is to activate silent paternal genes, including attempts to reactivate the paternal *Ube3a* allele in mice through gene therapy, using the topoisomerase inhibitor topotecan [12], or using ASOs to interfere with RNA to combat SNHG14 [13]. However, the toxicity and side effects of the drug are non-negligible. At the same time, interfering RNA has a short duration and cannot permanently activate the expression of paternal *UBE3A*, while the safety of gene therapy is still negotiating (Wolter et al., 2020). The lack of effective treatment options highlights the need to understand AS pathogenesis and downstream targets of UBE3A. The answers to these questions may lead to the development of new therapies.

In this work, we used RNA-seq to analyse the differential expression levels of RNA in the hippocampus of UBE3A-deficient mice and WT mice and performed other relevant experiments. The results indicated that UBE3A could act as a coactivator to regulate the RA signalling pathway. When UBE3A was lost, the downstream target gene expression levels were significantly decreased (Figure 4D). Perhaps this finding can provide new insights into the treatment of Angelman syndrome. Researchers may apply drugs that increase the overall transcription level to upregulate the expression of target genes in the RA signalling pathway, which may have a therapeutic benefit.

It is also important to note the limitations of our current research. Our experimental study does not rule out additional mechanisms that UBE3A function as E3 ligase regulating other substrates involving brain development (Greer et al., 2010; Sun et al., 2019). Therefore, the roles of UBE3A would need to be explored in follow-up study.

In summary, our work revealed a novel role of UBE3A in RA signalling regulation. Considering the great potential of chemicals and compounds already in the clinical trial related to RA signalling, our findings might facilitate the development of mechanism-based therapeutics for AS patients in the near future.

## Data Availability Statement

The datasets presented in this study can be found in online repositories. The names of the repository/repositories and accession number(s) can be found below: NCBI GEO GSE168889.

## Ethics Statement

The animal study was reviewed and approved by the Institutional Animal Care and Use Committee (IACUC) at the Center for Excellence in Molecular Cell Science, CAS.

## Author Contributions

LC, XG, and RH designed and supervised the whole UBE3A project. MF and YL performed most of the experiments. JR performed the data analysis. XG and MF drafted the manuscript. All authors read and approved the manuscript.

## Conflict of Interest

The authors declare that the research was conducted in the absence of any commercial or financial relationships that could be construed as a potential conflict of interest.

## Footnotes

1 http://www.genemania.org

## Supplementary Material

Supplementary Figure 1 The interaction network analysis of UBE3A obtained from GeneMANIA. The interaction types were exhibited as indicated in the network legend.

Supplementary Figure 2 UBE3A interacts with RARα in non-ubiquitination manner. **(A)** UBE3A binds medium region of RARα. The truncated RARα was purified by a GST tag and incubated with UBE3A-His protein for 4 h in GST pulldown buffer. GST; GST-RARα-1-87; GST-RARα-88-153; GST-RARα-153-462. **(B)** UBE3A did not affect the ubiquitination level of RARα. HEK-293T cells were transfected with His-UB, RARα-HA and Myc-UBE3A or Myc-UBE3A-C843A constructs. Ubiquitination-conjugated proteins were enriched using Flag beads and detected using an anti-HA antibody.

Supplementary Table 1 The list of genes most related to UBE3A.

Supplementary Table 2 Differentiated expressed gene list in RNA-seq.

Supplementary Table 3 The list of RA-regulated genes.

Supplementary Table 4 Overlapping genes.

Supplementary Table 5 Sequences of the siRNAs for UBE3A.

Supplementary Table 6 Sequences of the primers used in RT-qPCR.
