# Supplementary figures and images for: Epilepsy-Associated UBE3A Deficiency Downregulates Retinoic Acid Signalling Pathway
Source: Front Genet. 2021 Apr 28;12:681295. doi: 10.3389/fgene.2021.681295 (PMC8113777; doi:10.3389/fgene.2021.681295)

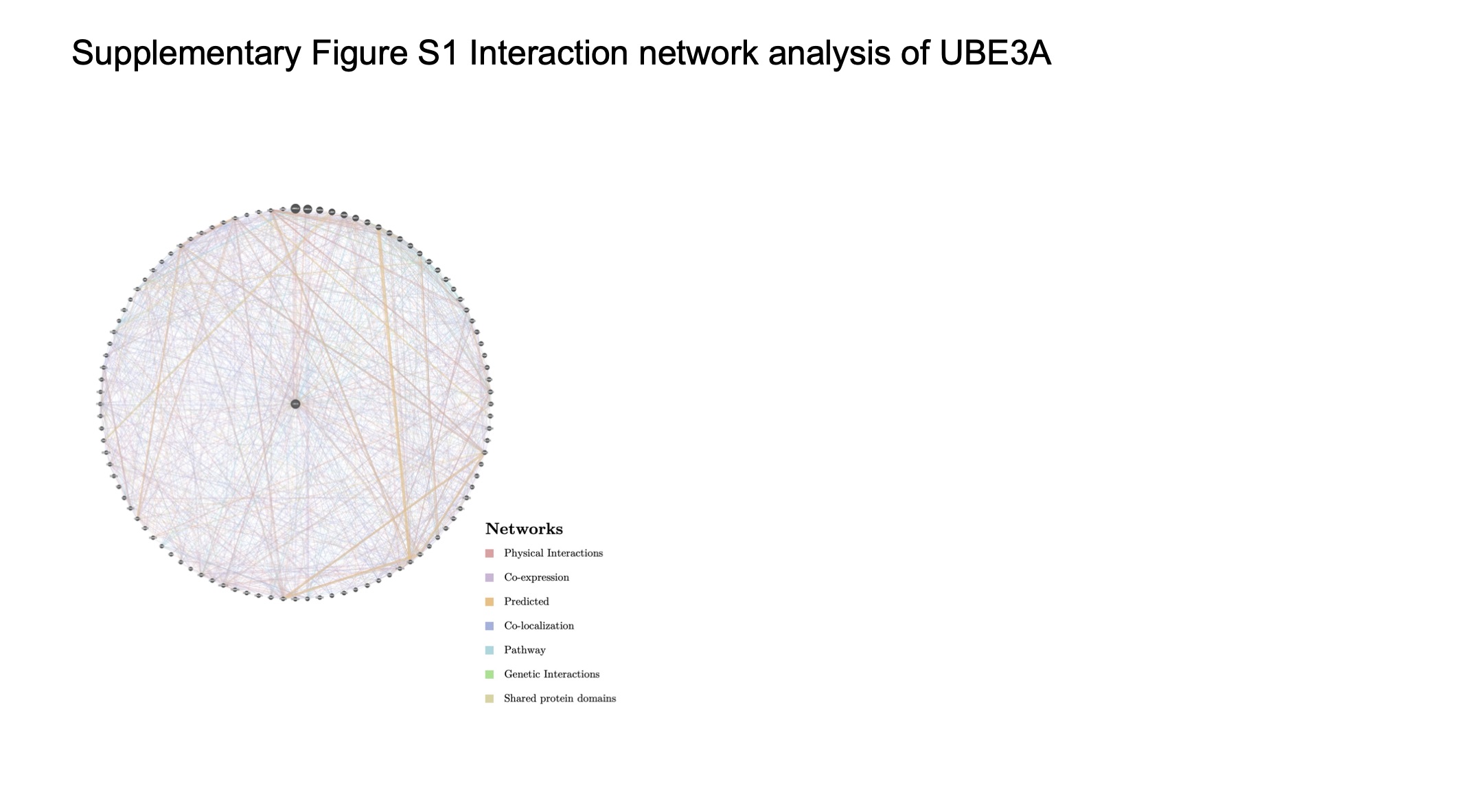

Supplement: Supplementary Figure 1 — The interaction network analysis of UBE3A obtained from GeneMANIA. The interaction types were exhibited as indicated in the network legend. [file Image_1.jpg]

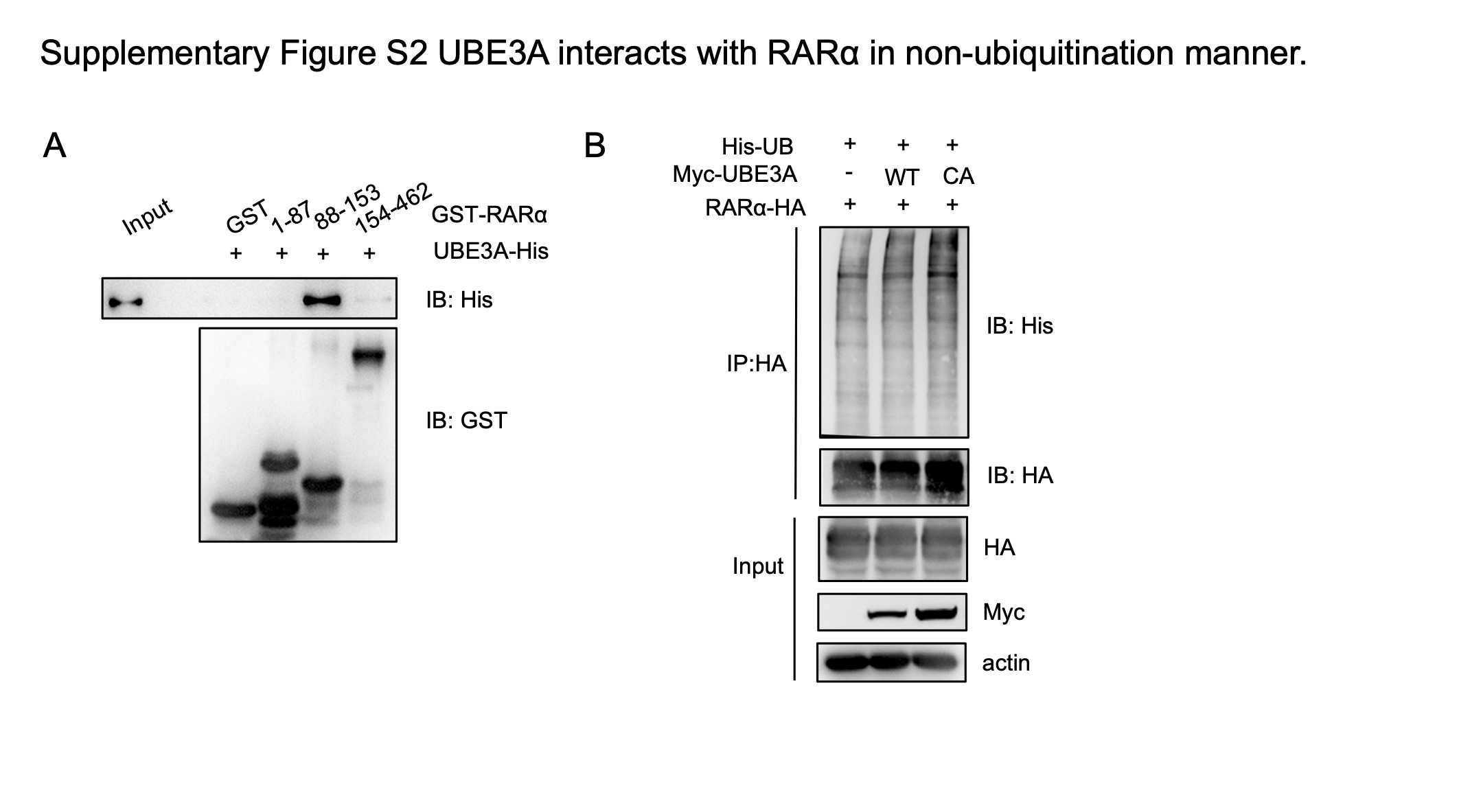

Supplement: Supplementary Figure 2 — UBE3A interacts with RARα in non-ubiquitination manner. (A) UBE3A binds medium region of RARα. The truncated RARα was purified by a GST tag and incubated with UBE3A-His protein for 4 h in GST pulldown buffer. GST; GST-RARα-1-87; GST-RARα-88-153; GST-RARα-153-462. (B) UBE3A did not affect the ubiquitination level of RARα. HEK-293T cells were transfected with His-UB, RARα-HA and Myc-UBE3A or Myc-UBE3A-C843A constructs. Ubiquitination-conjugated proteins were enriched using Flag beads and detected using an anti-HA antibody. [file Image_2.JPEG]
